# Phasing in on the cell cycle

**DOI:** 10.1186/s13008-018-0034-4

**Published:** 2018-01-25

**Authors:** Steven Boeynaems, Peter Tompa, Ludo Van Den Bosch

**Affiliations:** 1 Department of Neurosciences, Experimental Neurology and Leuven Research Institute for Neuroscience and Disease (LIND), KU Leuven-University of Leuven, 3000 Leuven, Belgium; 2 Laboratory of Neurobiology, VIB, Center for Brain and Disease Research, 3000 Leuven, Belgium; 3 Department of Genetics, Stanford University School of Medicine, Stanford, CA 94305 USA; 4 VIB, Center for Structural Biology (CSB), Vrije Universiteit Brussel (VUB), Brussels, Belgium; 5 Institute of Enzymology, Research Centre for Natural Sciences of the Hungarian Academy of Sciences, Budapest, Hungary

**Keywords:** Protein phase separation, Oncogenic fusion, Protein aggregation, Cancer, Stress granules, Nucleolus, Centrosome

## Abstract

Just like all matter, proteins can also switch between gas, liquid and solid phases. Protein phase transition has claimed the spotlight in recent years as a novel way of how cells compartmentalize and regulate biochemical reactions. Moreover, this discovery has provided a new framework for the study of membrane-less organelle biogenesis and protein aggregation in neurodegenerative disorders. We now argue that this framework could be useful in the study of cell cycle regulation and cancer. Based on our work on phase transitions of arginine-rich proteins in neurodegeneration, via combining mass spectroscopy with bioinformatics analyses, we found that also numerous proteins involved in the regulation of the cell cycle can undergo protein phase separation. Indeed, several proteins whose function affects the cell cycle or are associated with cancer, have been recently found to phase separate from the test tube to cells. Investigating the role of this process for cell cycle proteins and understanding its molecular underpinnings will provide pivotal insights into the biology of cell cycle progression and cancer.

## Background

Compartmentalization is a key feature of life. The cell membrane defines the context of what is living and what is not. Yet, this is only the first stage of the spatial organization of living matter. Cells, and in particularly eukaryotes, are further divided in subcompartments termed organelles, each of them carrying out specific biochemical reactions. These organelles can be roughly divided in two classes: membrane-bound and membrane-less. Whereas the processes behind the formation of membrane-bound vesicles have been relatively well-studied, the biogenesis and properties of their membrane-less counterparts remained elusive.

Membrane-less organelles (e.g. the nucleolus, stress granules, …) often consist of protein and RNA. Yet how does a cell concentrate these biomolecules without a membrane barrier? In recent years the phenomenon of liquid–liquid phase separation was found to underlie the biogenesis of these compartments [1–9]. Multivalent interactions predominantly mediated by intrinsically disordered and low complexity domains drive the spontaneous demixing of the RNA binding proteins involved [1–10]. This demixing results in the formation of liquid-like protein droplets or protein hydrogels, and depends on specific in vitro conditions, such as concentration, salt and temperature [1–9]. Interestingly, such test-tube phases are highly reminiscent of cellular RNA granules, suggesting that protein phase transition might indeed be the physical basis of the biogenesis of membrane-less organelles [1, 7, 8, 11]. However, these dynamic test tube assemblies seem to spontaneously mature to a more solid-like state [7, 8, 12–14], suggesting that they could act as stepping stones towards protein aggregation. Indeed, membrane-less organelles have been suspected to serve this role in some neurodegenerative disorders [15, 16].

For example, amyotrophic lateral sclerosis (ALS) is an adult-onset neurodegenerative disorder characterized by the aggregation of RNA binding proteins (RBPs) in the central nervous system [17]. Based on similarities in protein content between RBP aggregates and stress granules, these membrane-less organelles have been suggested as seeds for pathological aggregation of RBPs in patients [15, 16]. However, why these proteins undergo this liquid-to-solid switch during aging is unknown. Rare disease mutations found in some of these proteins make them more aggregation-prone [7, 8, 13], yet these cases do not explain why the wildtype proteins also aggregate in the majority of ALS cases. Hexanucleotide repeat expansions in the *C9orf72* gene are the most common genetic cause of ALS [18, 19], and recent evidence points at unconventional dipeptide repeat (DPR) peptides derived from the expanded repeat RNA as a major pathogenic species in the disease [20–25]. While five different DPRs are formed, two arginine-rich ones (i.e. glycine–arginine and proline–arginine, or GR and PR), are highly toxic in disease models [20–25]. We and others have recently found that these arginine-rich DPRs can phase separate in the presence of RNA [25]. Additionally, these toxic peptides promote a liquid-to-solid switch of stress granules in cells [25, 26]. Hence, providing an explanation why the involved RBPs start to aggregate in the most common genetic form of the disease.

## Main text

### Proteins involved in cell cycle phase separate in vitro

**Fig. 1:**
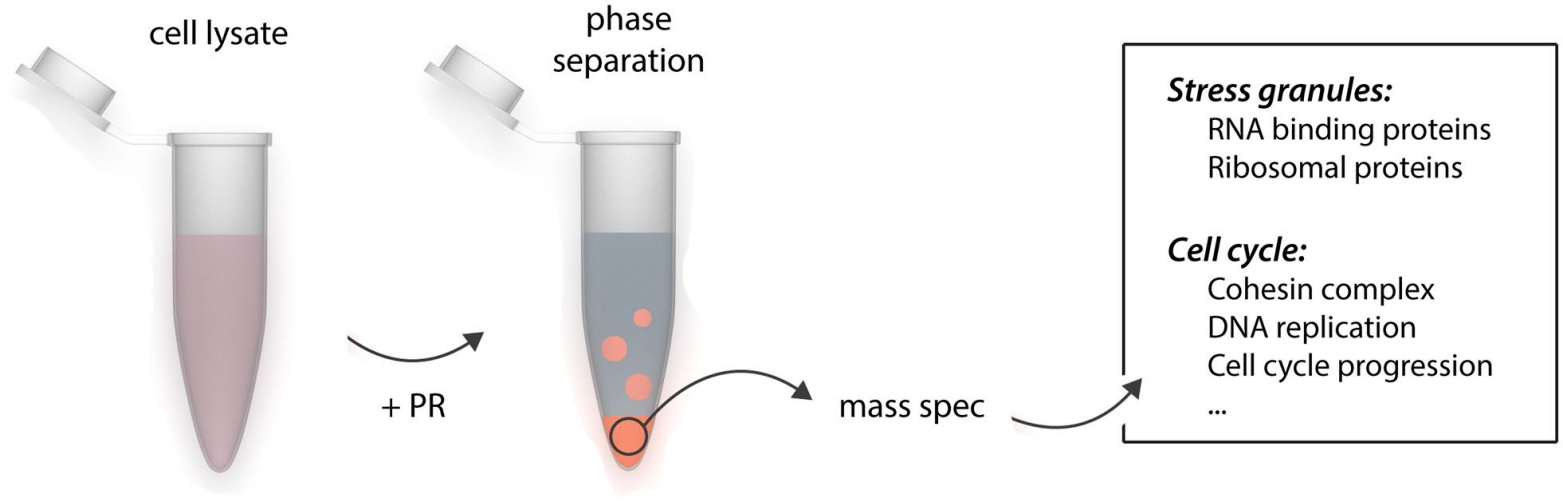
Identification of the phase separating proteome. Cleared cell lysate was incubated with poly-PR peptide to induce phase separation of cellular proteins. Phase separated proteins were precipitated by mild centrifugation and subjected to mass spectrometry. Identified proteins included stress granule factors and other membrane-less organelle components, but surprisingly as well proteins annotated as implicated in the regulation of the cell cycle

We have extensively characterized our PR-RNA granule system and argue its usefulness as a test tube model for protein phase separation [26]. To identify cellular proteins prone to arginine-mediated phase separation, we performed mass spectrometry (MS) [27] (see Fig. 1). We incubated soluble HeLa cell lysate, cleared from the insoluble fraction, with PR peptide. This resulted instantaneously in phase separation of PR with cellular proteins, observed as a clouding of the sample. Through mild centrifugation we collected these phase separated proteins into a pellet, which we showed was dependent on both weak liquid-like and more stable solid-like interactions [27]. Such a stable core/liquid shell topology is also observed for membrane-less organelles in living cells [11]. We identified 874 proteins in our sample, which were enriched for RBPs and proteins involved in stress granule metabolism, hereby confirming our observations from cells where we found that PR targets and perturbs stress granules [27].

**Fig. 2:**
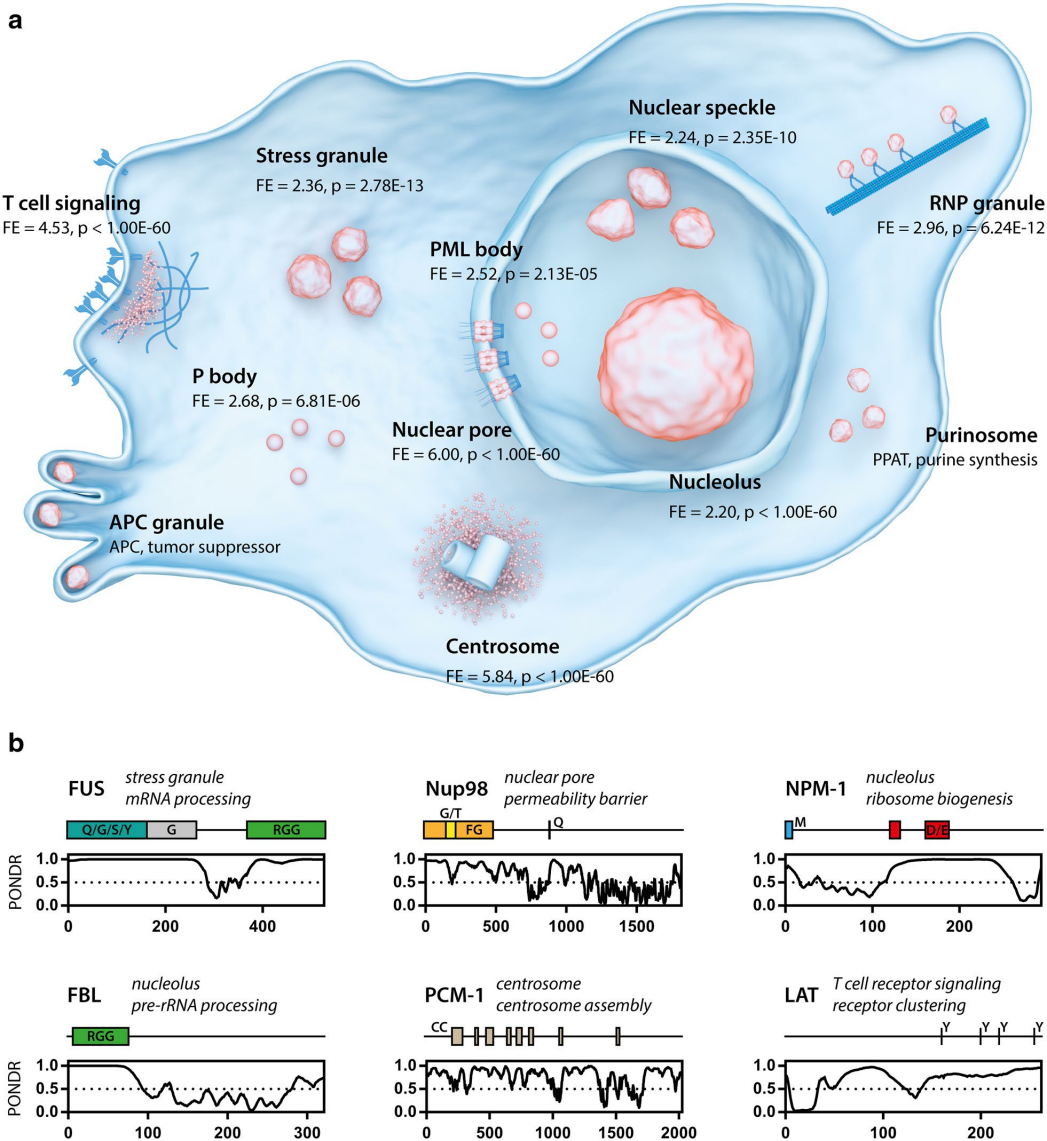
Proteins regulating or affecting the cell cycle are involved in cellular phase separations. **a** Overview of different membraneless organelles (orange). The fold enrichment of cell cycle proteins (GO:0000278) is shown for each organelle for which the protein content was available. T cell signaling (GO:0050852) [40], stress granule [11], nuclear speckle (GO:0016607), RNP granule (GO:0035770), PML body [73], P body (GO:0000932), nuclear pore (GO:0005643) [74], nucleolus [75], centrosome (GO:0005813) [76]. APC granules and purinosomes were positive for cell cycle proteins APC [77] and PPAT [78] respectively. **b** Examples of cell cycle proteins found in membrane-less organelles which can undergo phase separation (see Table 1). PONDR disorder prediction plots are shown, indicating prevalence of disordered regions in these proteins (score > 0.5). Coiled coil (CC) and low complexity domains (letters indicate overrepresented amino acids) are also indicated. Phosphotyrosine residues necessary for receptor clustering are indicated for LAT

Interestingly though, further analysis in follow-up work showed that our PR dataset is also highly enriched for proteins involved in the regulation of the cell cycle (GO:0000278; fold enrichment = 3.83, p = 2.93E−23, Fisher Exact Bonferroni). In Table 1 we provide an overview of some cell cycle proteins (KEGG pathway: hsa04110) that we identified in our MS experiment. This finding suggests that phase separation could also play a role in the regulation of this process. A common feature of proteins that undergo phase separation is structural disorder [10]. Indeed, proteins involved in cell cycle regulation are on average more disordered (IUPred score; median fold change = 1.69, p < 1.00E−4, Mann–Whitney) compared to the proteome. Besides being mostly intrinsically disordered, phase separating proteins also often show low sequence complexity, as exemplified by prion-like domains [28] (rich in uncharged polar amino acids and glycine) and arginine-rich domains [29]. Again, cell cycle regulatory proteins are enriched for both prion-like domains (fold enrichment = 1.39, p = 4.90E−02, binomial test) and arginine-rich domains (≥ 6 R-motifs/protein; fold enrichment = 2.44, p = 9.33E−15, binomial test) compared to the proteome. Besides these typical protein characteristics, several proteins that are known to phase separate also affect the regulation of the cell cycle (Table 2). Additionally, proteins regulating or affecting (even indirectly) the cell cycle are enriched in several known membrane-less organelles (Fig. 2a). Interestingly, some of these organelles also have been shown to dynamically change over the course of the cell cycle, as exemplified by the nucleolus, purinosome and centrosome [30–32]. Given that we found numerous cell cycle proteins in our MS dataset, and based on their physical characteristics and targeting to membrane-less compartments, we argue that there could be a previously unappreciated role for protein phase transition in the regulation and execution of the cell cycle.

**Table 1:** Cell cycle proteins identified in the mass spec experiment

Gene name	Protein name	Role in cell cycle
CDC5L	Cell division cycle 5-like	Cell cycle progression
CDC37	Cell division cycle 37	Cell cycle progression
CDC42	Cell division cycle 42 homolog	Kinetochore complex
CDC73	Cell division cycle protein 73 homolog	Cell cycle progression
CDK11A	Cyclin-dependent kinase 11A	Cell cycle progression
CDK11B	Cyclin-dependent kinase 11B	Cell cycle progression
CUL4A	Cullin-4A	Cell cycle progression
CUL4B	Cullin-4B	Cell cycle progression
HDAC1	Histone deacetylase 1	Cell cycle progression
MAD1L1	Mitotic spindle assembly checkpoint protein MAD1	Spindle-assembly checkpoint
MCM2	DNA replication licensing factor MCM2	DNA replication initiation
MCM3	DNA replication licensing factor MCM3	DNA replication initiation
MCM4	DNA replication licensing factor MCM4	DNA replication initiation
MCM5	DNA replication licensing factor MCM5	DNA replication initiation
MCM6	DNA replication licensing factor MCM6	DNA replication initiation
MCM7	DNA replication licensing factor MCM7	DNA replication initiation
PCNA	Proliferating cell nuclear antigen	Control of DNA replication
PRKDC	DNA-dependent protein kinase catalytic subunit	Sensor for DNA damage
RAD21	Double-strand-break repair protein rad21 homolog	Cohesin complex
SMC1A	Structural maintenance of chromosomes protein 1A	Cohesin complex
SMC2	Structural maintenance of chromosomes protein 2	Cohesin complex
SMC3	Structural maintenance of chromosomes protein 3	Cohesin complex
SMC4	Structural maintenance of chromosomes protein 4	Cohesin complex
YWHAB	14-3-3 protein beta/alpha	Cell cycle progression
YWHAE	14-3-3 protein epsilon	Cell cycle progression
YWHAG	14-3-3 protein gamma	Cell cycle progression
YWHAH	14-3-3 protein eta	Cell cycle progression
YWHAZ	14-3-3 protein zeta/delta	Cell cycle progression

**Table 2:** Phase separated proteins whose function affects the cell cycle

Protein name	Effect on cell cycle		Phase separation
Nucleolus			
FIBL	Knockdown reduces cell growth [57]		[12]
NPM-1^a^	Role in tumorigenesis [58]		[12]
Stress granule			
eIF4G2	Knockdown induces apoptosis and impairs proliferation [59]		[9]
FUS^a^	Knockdown impairs cell proliferation [60]		[6, 8, 9, 51]
EWS^a^	Knockdown induces apoptosis and impairs proliferation [61]		[2]
hnRNPA1	Knockdown induces cell cycle arrest [62]		[7, 9]
TDP-43	Overexpression induces cell cycle arrest [63]		[64]
TIA-1	Knockdown promotes cell proliferation [65]		[9]
Centrosome			
PCM-1	Involved in cell cycle progression [66]		[67]
Purinosome			
PPAT	Regulates growth rate via de novo purine biosynthesis [68]		[69]
Nuclear pore			
Nup98^a^	Regulates expression cell cycle genes [70]		[71]
T-cell receptor			
LAT	Regulates T-cell activation and proliferation [72]		[40]

^a^ Highlights protein found as oncogenic fusion proteins [46–49, 52]

### How could membrane-less organelles be involved in the cell cycle and cancer?

We have shown that proteins involved in the regulation of the cell cycle are enriched for features commonly associated with phase separation, and many of these proteins are components of membrane-less organelles. This begs the question how membrane-less organelles could be functionally implicated in the regulation of the cell cycle? What purpose do they serve, and are they altered in cancer?

Compartmentalization has been known to serve different functions: (1) Catalyzing biochemical reactions by concentrating reaction compounds, (2) shielding components from each other by localization in different compartments, (3) storage of biomolecules for later use, and (4) signal amplification. First, the laws of chemistry dictate that at higher concentrations of the reaction components, the reaction efficiency will increase. It seems that exactly this is the function of the pericentriolar material. This membrane-less organelle concentrates tubulin monomers, which are subsequently efficiently nucleated and grown into microtubules [33]. Secondly, chromatin architecture is known to be linked to the cell cycle [34]. Recently, two groups reported that heterochromatin domains form also by a process of phase separation [35, 36], showing that this process is key in regulating chromatin architecture. Additionally, during mitosis a specific disordered protein associates with the compacted DNA and acts as a biological surfactant to prevent the condensed sister chromatids from sticking together [37]. Upon nuclear envelope formation however, another helical protein exactly does the opposite, by crosslinking the condensed DNA to ensure the formation of one nucleus [38]. These findings show that phase separation is key in the organization of DNA over the cell cycle. Thirdly, upon quiescence due to nutrient-limiting conditions the cell’s proteasomes will assemble in cytoplasmic proteasome storage granules. Upon reentry into the cell cycle, these granules disassemble and the proteasome complexes translocate back to the nucleus to carry out their function [39]. Lastly, protein phase separation can also promote signal amplification. One of the best examples illustrating this process is the phase separation of T-cell receptors upon stimulation. Their phase separated intracellular domains concentrate signaling molecules to generate a robust signaling response activating cellular differentiation programs [40].

Besides the regulatory functions of membrane-less organelles and protein phase separation in processes associated with the cell cycle, they are sometimes also altered in cancer. For example, different cancers have a reported increase in stress granules and paraspeckles. As the increased number of these membrane-less organelles has been linked to a poor prognosis for survival [41–43], it makes them an interesting therapeutic target. Additionally, aggregation of different tumor suppressor proteins, including p53, results in their loss of function and is a major mechanism in cancer [44]. Compounds preventing its aggregation have been successful in preclinical animal models [45], indicating that indeed protein phase transitions could be viable therapeutic options.

### Phase separation is a novel mechanism of oncogenic fusion proteins

Interestingly, several of the disordered proteins prone to phase separation are also known to be involved in cancer-related fusion events (see Table 2). The most relevant examples are FUS and EWS, which are also components of stress granules and aggregate in ALS [17]. For example, FUS is involved in the chimera FUS-CHOP in liposarcomas [46]. EWS on the other hand is found as an EWS–FLI1 fusion causal for sarcomas and leukemias [47], and as an EWS–ATF1 fusion in melanomas [48]. Another notable example is nucleophosmin (NPM-1), a key component of the nucleolus, which is also part of the NPM-ALK fusion product in non-Hodgkin’s lymphoma [49]. Lastly the nuclear pore protein NUP98 is found in many oncogenic fusions involved in leukemias [50].

Several observations confirm that the correlation between phase separation and fusion proteins is more than coincidental. There are three essential features of proteins that prevail in both classes, which suggests a causative link between the two cellular processes. First, phase separating proteins have a high level of structural disorder [5, 51], which is also the case with oncogenic fusion proteins [52]. Second, phase separation relies on transient and multivalent protein–protein interactions [5], and the same principle holds true for oncogenic fusions. For example, activation of the oncogenic NPM-ALK chimera requires transient oligomerization mediated by the NPM segment [49], and coiled-coil interaction motifs are central to the autoactivation of many other oncogenic fusions [52]. Exactly, such protein–protein interaction domains are enriched in proteins undergoing phase separation (see Fig. 2b). Third, a recurrent feature of cellular phase separation is the presence of RNA and RNA-binding proteins [6, 16]. Again, oncogenic fusion proteins are also significantly enriched in RNA- and DNA-binding domains [52, 53].

It is believed that these disordered domains act as transcription activation domains, and hence drive gene expression where the fusion protein interacts with the DNA [51, 52]. Recent evidence has shown that the transcriptional activation potential of these disordered domains is directly correlated to their ability to phase separate. Kwon et al. [54] generated synthetic fusions of the FUS low complexity domain and fused it to a GAL4 DNA binding domain. By making point mutations which interfere with the hydrophobic interactions required for phase separation, the researchers not only perturbed hydrogel formation in the test tube, but also with transcriptional activity of the fusion protein in cells [54]. Compellingly, a new study found exactly the same mechanism of action in relevant EWS–FLI fusions observed in patients [55]. Also in this study there was a direct correlation between the potential to phase separate and to activate transcription by these disordered domains [55]. Indeed, several groups have shown that the C-terminal domain of RNA polymerase II has a strong affinity for phase separated disordered domains [51, 54, 56], illustrating how local phase separation can recruit the transcriptional machinery to distinct genomic regions and drive the oncogenic transformation of cells.

## Conclusions

In the last few years, the concept of protein phase separation has taken the field of cell biology by storm. This physical phenomenon provides a clear framework for the understanding of membrane-less organelle biogenesis. Moreover, this insight has given us a new view on protein aggregation in the context of human disease, and more specifically in neurodegenerative disorders. In our previous experiments on the role of protein phase transition in the pathogenesis of ALS, we developed a test tube model for the study of this process. Using MS, this simple model allowed us to perform a proteome-wide search for proteins which could undergo phase separation. Unexpectedly, we identified numerous proteins which were directly involved in the cell cycle or its regulation. This finding strongly suggests that protein phase separation could be at play in cell cycle regulation and associated diseases such as cancer.

To test this hypothesis, we examined in this commentary the physical characteristics of proteins involved in cell cycle regulation, and combined this with new insights from recent studies in the phase transition field. Not only do proteins involved in cell cycle regulation have the right physical characteristics for phase separation, we do know they are actually enriched in cellular membrane-less organelles. Lastly, we suggest different mechanisms of how membrane-less organelle formation and related processes could be functionally involved in cell cycle regulation and misregulation in cancer. We postulate that there is increasing evidence for such a functional involvement which warrants further experiments to uncover its full extent. To conclude, we would like to argue that the framework of protein phase separation could be useful to the study of the cell cycle in health and disease, and may guide the development of novel therapeutic approaches.

## Abbreviations

ALS: amyotrophic lateral sclerosis
DPR: dipeptide repeats
GR: glycine–arginine
MS: mass spectrometry
PR: proline–arginine
RBP: RNA-binding protein
